# Supplementary figures and images for: Risk factors and related miRNA phenotypes of chronic pain after thoracoscopic surgery in lung adenocarcinoma patients
Source: PLoS One. 2024 Mar 14;19(3):e0297742. doi: 10.1371/journal.pone.0297742 (PMC10939217; doi:10.1371/journal.pone.0297742)

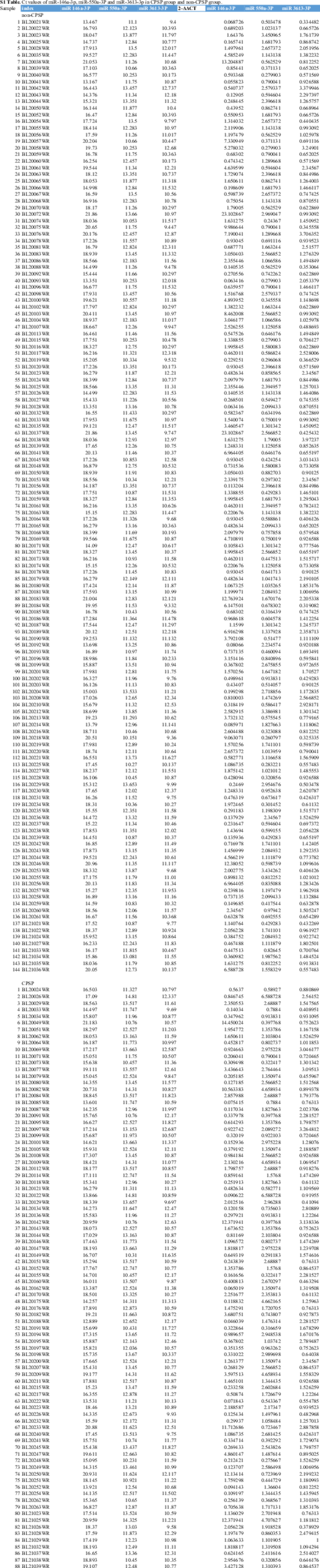

Supplement: S1 Table — (TIF) [file pone.0297742.s001.tif]
