# Supplementary material for: Risk factors and related miRNA phenotypes of chronic pain after thoracoscopic surgery in lung adenocarcinoma patients
Source: PLoS One. 2024 Mar 14;19(3):e0297742. doi: 10.1371/journal.pone.0297742 (PMC10939217; doi:10.1371/journal.pone.0297742)
Supplement: Attachment — Submitted filename: Response to Reviewers.doc [file pone.0297742.s002.doc]

August 20, 2023

Dr. Emily Chenette

Editor-in-Chief

PLOS ONE

Dear Dr. Chenette:

Thank you very much for your e-mail dated Jun 28 2023 providing the reviewers' comments on our submission (ID：PONE-D-23-13874) entitled “Risk factors and related miRNA phenotypes of chronic pain after thoracoscopic surgery in lung adenocarcinoma patients”. We appreciate editor and reviewers very much for the positive and constructive comments on our manuscript. Meanwhile, we are very grateful to you for giving us an opportunity to revise our manuscript.

We have carefully considered the comments and modified several parts of the manuscript which marked in highlights changes made to the original version. We are sorry for our mistake and lack of understanding of the mismatch between ‘Funding Information’ and ‘Financial Disclosure’ mentioned in the editor's letter. We have checked the Funding source, number and recipient in the ‘Funding Information’ section and confirmed that they are correct. Meanwhile, we found that the initial information submitted in ‘Financial Disclosure’ section was incomplete. Therefore, we need to supplement the following statements in the 'Financial Disclosure' section: This work was supported by Quanzhou City Science & Technology Program of China [Grant no: 2019N105S]. And, the funders had no role in study design, data collection and analysis, decision to publish, or preparation of the manuscript.

Thanks again to the hard work of the editor and reviewers! Looking forward to hearing from you.

Yours sincerely,

Hefan He

Department of Anesthesiology, the Second Affiliated Hospital of Fujian Medical University, Quanzhou 362000, China

Phone: +8615860905262

Email: 15860905262@fjmu.edu.cn

General response: We thank Editor and Reviewers for both the time and the insightful comments. We have carefully taken into consideration all the comments, and revised the manuscript substantially by adding analysis and clarifying content to make it more scientific and innovative. We have addressed all questions in a point-by-point manner, as shown below.

**Journal requirements:**

When submitting your revision, we need you to address these additional requirements.

1. Please ensure that your manuscript meets PLOS ONE's style requirements, including those for file naming.

**Response:** Thanks for your reminder and the handy link. We have read PLOS ONE Formatting guidelines carefully and made sure that our manuscript meets PLOS ONE's style requirements as the manuscript shows.

2. We note that the grant information you provided in the ‘Funding Information’ and ‘Financial Disclosure’ sections do not match.

When you resubmit, please ensure that you provide the correct grant numbers for the awards you received for your study in the ‘Funding Information’ section.

**Response:** We are very sorry for our negligence of providing the wrong the message on the 'Financial Disclosure' section. We have corrected the Financial Disclosure to make the grant information consistent in the 'Funding Information' and 'Financial Disclosure'. And we have updated the statement in our cover letter.

Reviewers’ comments

Reviewer#1

**Comment No.1:** The number of citations and the selected citations do not adequately reflect the current state of research. The authors should consider current literature. **Response:** We agree wholeheartedly that our manuscript needs to add more current literature. In the revised manuscript, we have updated the literature by replacing the older citations and adding some more recent literature to our analysis, such as Sabina S et al. (2022), Jin J et al. (2022), Chen WC et al. (2022), Jiang M et al. (2022), Zhang Y et al. (2022), Pellegrino A et al. (2023), Chen J et al. (2022) and Shi, Y et al. (2018), Phạm TL et al. (2020).

**Comment No.2:** A description of how pain was measured and classified is completely missing. There are several accepted standards in research. Which of these was used and what the results. This data is needed to be added.

**Response:** We are very grateful to the Reviewers for their constructive suggestions. We only studied whether patients had CPSP after surgery, Thus, the patients were inquired only about the worst pain intensity, pain location and their current analgesic use. We have added Chinese validated version of the BPI-SF (Brief Pain Inventory Short Form) to assess pain intensity. And the description of how pain was measured and classified has been completed. (Page 6, lines 101–116)

**Comment No.3:** Furthermore, a correlation of these parameters with the miRNA profile should be done.

**Response:** We fully agree that a correlation analysis of the parameters should be done. We performed collinearity diagnosis on all the covariables included in the regression analysis and found that the tolerance＞0.2 and VIF＜5. It could be considered that there was no collinearity as shown in Table 5. (page 14, Table 5)

**Comment No.4:** The description of the methods is insufficient. E.g., page 7 line 123-124 "After an appropriate amount of TRIZOL ...". This is not a scientific description that allows repeating the experiments. For each experimental step, a detailed protocol should be given as stated in the Author guidelines.

**Response:** We are very sorry for our negligence of our experimental procedure description. We have already added the description for each experimental step. (page7-10, lines 130–188)

**Comment No.5:** Page 7: Construction of miRNA Library

1. Number of samples is missing

2. Quality control of each step is missing

3. Why was the miRNA not isolated from serum?

4. Which threshold for differentially expressed miRNA was chosen?

5. How was "possibly greater correlation with chronic pain" determined?

**Response:** （For questions 1 and 5）We are sorry that we may have not expressed clearly about the number of sample and the reason for the chosen miRNAs which were possibly greater correlative with chronic pain.

Actually, three white blood cell samples from each group treated with TRIzol were randomly selected and submitted to Shanghai Europe Easy Biomedical Technology Co., Ltd., to complete the construction of a small RNA library and high-throughput sequencing. (And one case found as unqualified in quality inspection was rejected.) (page8, lines 153–157)

The steps on how to select miRNAs which are more correlative with chronic pain are as follows: Firstly, we completed the construction of a small RNA library and high-throughput sequencing. Secondly, we detected a total of 24 differentially expressed miRNAs (p-value<0.05&|log2FC|>1). Thirdly, we selected three miRNAs with obvious differential expression through atlas analysis and literature search [6,8]. And the above expression has been placed in the ‘Construction of a small RNA library and high-throughput sequencing’ section. (page8, lines 153–164)

（For question 2）We fully agree that the quality control for each step should be supplemented. We have added quality control to the extracted total RNA (page8, lines 148–150,155-157) and the PCR detection (page9, lines 183–185).

（For question 3）It is really true as Reviewer mentioned that the miRNA we got was not from serum. Because the miRNA in serum may come from both intracellular and extracellular sources, such as red and white blood cells. The amount of miRNA directly extracted from cells will be more. Moreover, one of the mechanisms leading to chronic pain is inflammation, and white blood cells are closely related to inflammation, so we chose white blood cells to extract miRNA to study the expression difference between the two groups.

（For question 4）We are grateful to the Reviewer for the reminder. Actually, the threshold for differentially expressed miRNA was p-value<0.05 and |log2FC|>1. (page8, line 159).

**Comment No.6:** Page 8: qPCR

o Why was total RNA used for qPCR? There is a risk that the primers are not specific.

o The sequence of the reverse primer used is missing

o qPCR conditions are missing and a table containing the Ct values of the samples

**Response:** We very much appreciate the Reviewer’s valuable comments and suggestions. The design and synthesis of primer sequences for the three miRNAs were specific which can be found in Table 1 of the text. And the reverse primers came with kits, but we are sorry that the reverse sequences couldn’t be provided due to confidentiality of the other party. We have added complete qPCR conditions. (page9-10, lines 169–188) And the Ct values of all the samples have been provided in S1 Table.

**Comment No.7:** statistical analysis

o How was the necessary sample size (number of patients) calculated?

o Information on which patients were excluded / included

**Response:** We are very grateful to the Reviewer for the constructive suggestions. We have added detailed basis for sample size calculation, and list them separately in the "Sample size" section. (page10, lines 191–201) And we have placed the included / excluded criteria in the’ Patients and inclusion criteria’ section. (page4, lines 60–69)

**Comment No.8:** Results:

- The distribution of patients between the two groups is uneven e.g., the CPSP group contains more women.

- How was it determined that differences in clinical information did not affect the data? What mathematical models were used?

- Were the data analysed by further subdividing the groups e.g., male samples only, young patients vs older patients, etc.?

**Response:** Thanks for Reviewer’s questions. We aimed to explore the risk factors relating to chronic postsurgical pain (CPSP). And the patients were divided into two groups (non-CPSP and CPSP) according to the presence or absence of CPSP. Therefore, there existed some differences in clinical information which were the variables we were looking for. It is really true that the CPSP group contains more women, but there are no significant differences in sex between the two groups. If there was a significant difference, then we would include it in the regression analysis to determine the risk factor.

**Comment No.9:** Discussion:

- On page 13, line 240 it is stated that after surgery 37.9% of patients developed CPSP. What is the normal range?

- The discussion lacks any description of the biochemical function and pathways involved of the miRNAs. At least one GO annotation and appropriate discussion needs to be made

- Which miRNAs have been identified / described in patients with early adenoma carcinoma?

- Which miRNAs were identified / described in patients with chronic pain?

- The miRNA part of the discussion is a collection of known facts of the identified miRNAs but no discussion. The whole section needs to be revised.

**Response:** We very much appreciate the Reviewer’s valuable comments and suggestions. According to the literature, the incidence of CPSP after thoracic surgery is 25-75%. (Chen WC, Bai YY, Zhang LH, et al. Prevalence and Predictors of Chronic Postsurgical Pain After Video-Assisted Thoracoscopic Surgery: A Systematic Review and Meta-analysis. Pain , 2023,12(1):117-139. doi: 10.1007/s40122-022-00439-0.）And we have re-written this part according to the Reviewer’s suggestion. (page15,16,17)

Reviewer#2

**Comment:** The authors need to make it very clear that correlation does not mean causation - this is not clear.

Second, authors need to clarify whether the factors identified are correlated to each other or how the statistical/correlation analyses were performed and corrected for other co-variables.

**Response:** We very much appreciate the Reviewer’s valuable comments and suggestions. We fully agree that correlation does not mean causation. We have performed collinearity diagnosis on all the covariables included in the regression analysis and found that the tolerance＞0.2 and VIF＜5. It could be considered that there was no collinearity as shown in Table 5. (page 14, Table 5)
