# Supplementary material for: Risk factors and related miRNA phenotypes of chronic pain after thoracoscopic surgery in lung adenocarcinoma patients
Source: PLoS One. 2024 Mar 14;19(3):e0297742. doi: 10.1371/journal.pone.0297742 (PMC10939217; doi:10.1371/journal.pone.0297742)
Supplement: Attachment — Submitted filename: Response to Reviewers.doc [file pone.0297742.s003.doc]

November 17, 2023

Dr. Emily Chenette

Editor-in-Chief

PLOS ONE

Dear Dr. Chenette:

Thank you very much for your e-mail dated Oct 04 2023 providing the reviewers' comments on our submission (ID：PONE-D-23-13874R1) entitled “Risk factors and related miRNA phenotypes of chronic pain after thoracoscopic surgery in lung adenocarcinoma patients”. We appreciate reviewers very much for the positive and constructive comments on our manuscript. And we have carefully considered the comments and modified several parts of the manuscript which marked in highlights changes made to the original version.

We are very grateful to you for giving us an opportunity to revise our manuscript again. Thanks to the hard work of the editor and reviewers! Looking forward to hearing from you.

Yours sincerely,

Hefan He

Department of Anesthesiology, the Second Affiliated Hospital of Fujian Medical University, Quanzhou 362000, China

Phone: +8615860905262

Email: 15860905262@fjmu.edu.cn

General response: We thank Editor and Reviewers for both the time and the insightful comments. We have carefully taken into consideration all the comments, and revised the manuscript substantially by adding analysis and clarifying content to make it more scientific and innovative. We have addressed all questions in a point-by-point manner, as shown below.

Reviewers’ comments

**Reviewer#1 (No Comment)**

**Reviewer#2**

**General comments:** The figures are very rudimentary and no QC plots are shown. Analyses performed are very basic and further extensive analysis are required, for instance pathway analysis. Introduction is smaller than abstract and the reasoning behind study is not comprehensively described, hence, the rationale behind the study is not clear to me. Additionally, a diagram with the workflow and inclusion/exclusion, study design/timeline would be helpful.

**Response:**

We are very grateful to the Reviewer for the constructive suggestions. We have added ROC curve for the CPSP model, and found that the prediction probability for CPSP after VATS yield the area under the receiver operating characteristic curve of 0.781 (95% CI 0.718–0.844) (Figure 5.). Thanks to Reviewer 's reminder, we have added a diagram with workflow (Figure 3.).

We have revised the "Introduction" part and listed our research purpose at the end: The primary aim of this study was to identify independently predictors of CPSP after VATS. The second aim was to search for miRNA predictors of CPSP in peripheral blood, so as to provide directions for further research on the pathogenesis and development of CPSP.

We are very sorry for the lack of pathway analysis in our clinical study. In the study, we only found several clinical risk factors for the development of CPSP and one peripheral blood microRNA indicator, and the specific mechanism needs further study in the future.

**Main comments:**

**Comment No.1:** the authors conclude that preoperative BMI, preoperative expression level of miR-550a-3P in peripheral blood, preoperative history of chronic pain, and postoperative NRS score are risk factors for CPSP. However, do they mean all these factors need to be present? again, the authors do not clarify that correlation does not mean causation.
**Response:**

In this study, regression analysis was used to find several risk factors for CPSP, and collinearity diagnosis showed that there was no collinearity among these risk factors. They are independent for predicting the occurrence of CPSP, and do not need to present simultaneously.

We are very sorry that we failed to understand the point made by the reviewer in the first comments that correlation does not mean causation. Is it because we have not conducted further extensive analysis of miR-550a-3P to determine the causal relationship between miR-550a-3P and CPSP, but we presented some inappropriate expressions in the summary at the end of the "Discussion"? With this in mind, we have replaced " Inhibition of miR-550a-3P may serve as a novel therapeutic target for chronic pain after lung adenocarcinoma occurrence." with " The expression difference of miR-550a-3P may be one of the reasons for the genetic susceptibility to CPSP in patients with thoracoscopic lobectomy, which needs to be confirmed by further studies in the future.".

**Comment No.2:** following the questions above, The authors suggest that miR-550a-3P can be a target for post surgical pain. Would that by itself be able to prevent pain? To make this claim, authors need to perform statistical tests or experimental validation using in vitro or in vivo models.

**Response:** Given the complexity of post-surgical pain, many genes might contribute. MiR-550a-3P may only be one of the related factors. We regret that no corresponding experimental validation has been carried out.

**Comment No.3:** the authors mention that preoperative history of chronic pain is one of the factors for development of surgical pain. This is not novel and corroborated by several other studies in the literature. Would this factor by itself influence development of CPSP? Would targeting mir-550a-3P be sufficient to counteract all the other risk factors?

**Response:**

Logistic regression analysis showed that preoperative history of chronic is the risk factor for the occurrence of CPSP, and collinearity diagnosis on all the covariables included in the regression analysis showed there was no collinearity. We believe that the preoperative history of chronic pain may influence the development of CPSP by itself.

Given the complexity of post-surgical pain, many genes might contribute. In this study, we controlled some perioperative factors which may affect the occurrence of CPSP to search for CPSP susceptible population and find more closely related and more predictive genetic factors. So, expression difference of miR-550a-3P obtained in the study can well reflect the influence of genetic susceptibility on the occurrence of CPSP, but targeting mir-550a-3P alone would not be sufficient to counteract all the risk factors. (E.g. preoperative erector spinae plane block was performed to reduce the degree of acute postoperative pain. The difference of white blood cells before and after the operation did not have statistical significance could exclude the effect of postoperative infection on CPSP. All patients with early lung adenocarcinoma as their pathological diagnosis underwent video-assisted thoracoscopic lobectomy without lymph node dissection by the same group of surgeons. page 17)

**Comment No.4:** Wouldn't it be enough to know preoperative BMI and preoperative history of chronic pain and postoperative NRS to make a patient at risk of developing pain? If the rationale is that patients will need to go through surgery anyway because of cancer, and mir-550a could be a target for treating/preventing pain - then the authors needs to show actual data that backs up this claim. Additionally, this is not clear from the beginning why look at microRNAs. I do not see how relevant it is to know that mir-550a in peripheral blood is a risk factor for CPSP if the other risk factors are predictive by themselves.

**Response:** We are sorry that we did not clearly state the purpose of our research in "Introduction" at the beginning. And we have listed our research purpose at the end: The primary aim of this study was to identify independently predictors of CPSP after VATS. The second aim was to search for miRNA predictors of CPSP in peripheral blood, so as to provide directions for further research on the pathogenesis and development of CPSP.

**Comment No.5:** this sentence "Inhibition of miR-550a-3P may serve as a novel therapeutic target for chronic pain after lung adenocarcinoma occurrence" is not supported by evidence shown in the manuscript.

**Response:** We are grateful to the Reviewer for the reminder. We have replaced this sentence with " The expression difference of miR-550a-3P may be one of the reasons for the genetic susceptibility to CPSP in patients with thoracoscopic lobectomy, which needs to be confirmed by further studies in the future.".

**Comment No.6:** We also know from literature (see for instance Kehlet H, Jensen TS, Woolf CJ. Persistent postsurgical pain: risk factors and prevention. The lancet. 2006 May 13;367(9522):1618-25.) that genetic factors can play an important role. did the authors consider that and how do they address genetic factors in the context of the claims of this paper?

**Response:** Thanks for the paper provided by Reviewer. We read the review carefully, the article gives a brief description of the genetic factors. So far, several genes have been found to be associated with chronic pain, but the specific mechanism is still unclear.

**Comment No.7:** Authors do not address surgical technique, (or different surgeons?) - this can be a factor that influences development of pain as different techniques can target nerve fibers more.

**Response:** Thanks to the Reviewer for the reminder. We've added a description to "Methods". (Page 4, line 61…underwent thoracoscopic lobotomy without lymph node dissection by the same group of surgeons…)

**Comment No.8:** Inclusion/exclusion criteria do not mention pain immediately prior to surgery, which is a huge flaw of this study. Was this included under "4. Absence of nervous system dysfunction"? This is a very vague term and does not really clarify what factors contribute to it.

**Response:** We very much appreciate the Reviewer’s valuable comments and suggestions. We have added "No peripheral (somatic) or internal (visceral) chest pain before surgery" to the inclusion criteria, and replaced "Absence of nervous system dysfunction" with "Absence of peripheral neuropathy". (Page 4, lines 67,68)

**Comment No.9:** Authors did not look at pathway analysis associated with microRNAs, simply described previous literature in the Discussion.

**Response:** We very much appreciate the Reviewer’s comments. We have revised the discussion. Again, we are sorry about the lack of pathway analysis associated with microRNAs.

**Reviewer#3**

**Comment No.1:** Did you analyze in any way how these excluded patients could have influenced your results?

Why were complications such as pulmonary complication, which is a very broad definition, and re-hospitalization both exclusion criteria? You could expect that persistent pain with secondary effects also could be one of the pulmonary complications or leading to a complication and resulting in re-hospitalization. Please comment?

**Response:** We are very grateful to the Reviewers for their recognition of the manuscript and their constructive comments. The exclusion criteria have been reviewed and revised. (Page 4, lines 69-74) And the reasons for the selection of exclusion criteria were explained in the discussion. (Page 17, lines 295-301)

**Comment No.2:** Please check and comment or discuss the possible reference for miRNA’s: Sabina S, et al. Expression and Biological Functions of miRNAs in Chronic Pain: A Review on Human Studies. Int J Mol Sci. 2022 May 27;23(11):6016. doi: 10.3390/ijms23116016. PMID: 35682695; PMCID: PMC9181121. Could you explain why from all the possible miRNA’s that are in any way linked to chronic pain you chose miR-146-3P and the other 2? This should be described more precisely in the methods?

This may provide further support as to why you chose these specific mi-RNAs and why certainly not other MiRNAs.

**Response:** Thanks for the reviewer's suggestions. We have reviewed the reference for miRNA's: Sabina S, et al. and described the reasons for selecting mi-RNAs more precisely in the methods. (Page 9, lines 167-173)

**Comment No.3:** P4 regarding absence of nervous system disfunction: What is the definition? Were patients with preoperative well treated anxiety disorder, for instance with benzodiazepines also included or actually excluded?

**Response:** We are very sorry for our negligence, the expression of "absence of nervous system disfunction" in the inclusion criteria was wrong, we have changed it to "Absence of peripheral neuropathy". (Page 4, line 67)

**Comment No.4:** P6 regarding the statement about oxycodone sustained-release tablets: Besides the administration of these tablets twice a day, normally this is combined with the short acting opioids 4-6 times orally a day. Was this also normal procedure in your study?
P 6 last sentence “awake”: how was being awake scored?

**Response:** Thanks for Reviewer’s questions. In the study, patients were connected to intravenous analgesia pumps containing sufentanil after surgery, and oxycodone sustained-release tablets were given when patients still felt severe pain. No short acting opioids were added again. (Page 6, lines 101-104) Thanks to the reviewer's reminder, we have added the score of "awake" (with full Steward score). (Page 6, line 104)

**Comment No.5:** Were there any differences in final malignancy regarding the pathological final diagnoses regarding type of differentiation of the tumour, radicality of surgical resection, primary, metastatic. See for instance, differences between smokers, non-smokers who have quit smoking and the incidence of adenocarcinoma of the lung in these patients. Furthermore, there appears to be differences in smokers vs non-smokers in relation to the development of pain (CPSP) and gene up- or down regulation. How did this may have influenced your results?
P10 table 2 did the patients have a history of smoking or quit smoking or were non-smokers?

**Response:** Thanks for the reviewer's reminder and questions. Yes, of all the observed patients, 5 patients had different results in the final pathological diagnosis and were excluded. For clarity, we have added a separate item to the exclusion criteria for it. (Page 4, lines 70,71)

Indeed, it has been reported in the literatures that smoking status may be associated with CPSP. We re-included smoking history in the univariate analysis, but found no statistical difference between the CPSP group and non-CPSP group (P=0.212). Therefore, we no longer included smoking history in the regression analysis. (Table 2)

**Comment No.6:** Operation duration may be a surrogate for complexity of the procedure or tissue damage with p 0.09. Could this be related, actually the result of not being significant, to study power? Please discuss?
Were any other parameters that possibly display tissue damage or host response (e.g. inflammation) such as differences in CRP on days 1 or 2 among groups measured as you only illustrate white blood cell counts here? This is very scarse.

**Response:** We are very grateful to the Reviewer for the valuable comments. Considering that operation duration is likely to be related to CPSP, we adjusted the P-value limit (changed to P＜0.10) and included operation duration in regression analysis. Analysis results showed that operation duration was not a risk factor for CPSP(P=0.221). (Pages 14-16, lines 261-264, Table 6)

As mentioned by Reviewers, CRP could display inflammation. And many related studies have also conducted comparative analysis on it. But unfortunately, we missed this one in our study, which is the deficiency.

**Comment No.7:** See regarding molecular differences within the adenocarcinoma of the lung, among others, e.g., Solis LM, et al. Histologic patterns and molecular characteristics of lung adenocarcinoma associated with clinical outcome. Cancer. 2012 Jun 1;118(11):2889-99. doi: 10.1002/cncr.26584. Epub 2011 Oct 21. PMID: 22020674; PMCID: PMC3369269. There seems to be a difference and also in outcome? Did you take this histologic difference into account?

**Response:** Thanks for the literature recommended by reviewers, we have read it carefully. Whether histologic patterns and molecular characteristics of lung adenocarcinoma are related to the occurrence of CPSP is worthy of further study and analysis. At present, according to exclusion criteria 3, 4, and 5, we have excluded patients with poor prognosis or who require special treatment.

**Comment No.8:** And also among others regarding smokers and pain e.g. Oh TK, et al. Relationship between pain outcomes and smoking history following video-assisted thoracic surgery for lobectomy: a retrospective study. J Pain Res. 2018 Apr 6;11:667-673. doi: 10.2147/JPR.S157957. PMID: 29670393; PMCID: PMC5896682. How was this aspect present in your population?
See also Yoon S, Hong W, Joo H, et al. Long-term incidence of chronic postsurgical pain after thoracic surgery for lung cancer: a 10-year single-center retrospective study. Regional Anesthesia & Pain Medicine 2020;45:331-336. Here of a total of 3200 patients included in the analysis, 459 (14.3%) and 558 (17.4%) patients were diagnosed with CPSP within 3 and 36 months after surgery, respectively.
Were there any differences in duration of the ESP-block between patients or patients with failed blockade in relation to the development of pain during day 1-3 and the need for rescue medication. Please compare when possible lowest quartile vs highest quartile in the use of opioids or other analgesics?

**Response:** Thanks for the literatures recommended by reviewers. The first study showed that smoking history was not associated with postoperative pain scores, but was associated with morphine equivalent analgesics (mg) on postoperative days of 0-2. Consider that smoking history may be related to CPSP, we re-included smoking history in the univariate analysis, but found no statistical difference between the CPSP group and non-CPSP group (P=0.212). Therefore, we no longer included smoking history in the regression analysis. (Table 2)

Thanks for the reviewer's reminder and questions. The effect of nerve block may affect the development of postoperative pain. Therefore, we would test the exact effect of the ESP-block after completion. (Page 5, lines 92-95) And, failure to block would be excluded. (Exclusion criteria, page 4, line 74)

Thanks for the reviewer's suggestions. We compared the dosage of sufentanil, remifentanil and dexmedetomidine per kilogram during anesthesia. The results showed no statistical difference between CPSP group and non-CPSP group. (Table 2) Rescue medication was associated with postoperative NRS score. However, no correlation was allowed between the covariables included in the regression analysis, so we did not make additional comparison in rescue medication.

**Comment No.9:** P7 regarding the description to hemolyze the blood cells. Please describe procedure more in detail briefly? Could it be that blood was aspirated 5-10 times fast through a very small needle to induce hemolysis in combination with the anticoagulant?
Extraction of total RNA: here both brief description of the process and reference is needed?

**Response:** We very much appreciate the Reviewer’s reminder and have added the corresponding description. (Page 7, lines 137,138)
We are sorry that we did not quite understand the comments about the "Extraction of total RNA". We have described the whole process of Extraction of total RNA in the manuscript. (Pages 7-8, lines 144-157) And total RNA was extracted from white cells using mirVana^TM^ RNA Isolation Kit according to the manufacturer’s speciﬁcations. So, no more references were provided.

**Comment No.10:** You mention the resistin in adipose tissue in relation to pain development. See, e.g. Majchrzak M, et al. Increased Pain Sensitivity in Obese Patients After Lung Cancer Surgery. Front Pharmacol. 2019 Jun 14;10:626. doi: 10.3389/fphar.2019.00626. PMID: 31258474; PMCID: PMC6586739. What can be said by the possibility of genetic polymorphism altering sensitivity to pain and how it may have played an important role in CPSP development in the patients of your study regarding chronic pain might related both to obesity?
and differences in gene expression? Please comment and discuss? See: Hozumi J., et al. Resistin Is a Novel Marker for Postoperative Pain Intensity. Anesthesia & Analgesia 128(3):p 563-568, March 2019. | DOI: 10.1213/ANE.0000000000003363

**Response:** We very much appreciate the Reviewer’s valuable comments. And we have re-written this part according to the Reviewer’s suggestion. (page18, lines 319-326)

**Comment No.11:** Textual
Provide legend for figures and explain what should be seen for our readers and what is visible and focus on the most striking features and differences between groups in the figures. Furthermore, discuss these observations within the discussion in combination with chosen important references?

**Response:** Thanks for the reviewer's suggestion. We have presented two main resulting diagrams in the manuscript (Figure 4 and Figure 5), and explained the striking features and differences between groups in the figures. (page15, lines 266-274) And, we have revised the discussion according to the Reviewer’s suggestion.
